# The Principles and Practice of Surgery

**Published:** 1852-12

**Authors:** 


### EDITORIAL.

*The Principles and Practice of Surgery*, by JAMES MILLER, F. R. S. E., etc ; third American from the second and enlarged Edinburgh edition ; illustrated by two hundred and forty engravings on wood. Revised with additions, by F. W. SARGENT, M. D.: p. 751, 8vo. Philadelphia, Blanchard & Lea, 1852.

*The Principles and Practice of Surgery*, illustrated by 316 engravings on wood; by WM. PIRRIE, F. R. S. E., Regius Professor of Surgery in the Marischal College and University of Aberdeen, etc. : Edited, with additions, by JOHN NEILL, M. D., Surgeon to the Pennsylvania Hospital, etc.; p. 784. 8vo. Philadelphia, Blanchard & Lea, 1852.

*Lectures on the Principles and Practice of Surgery*, by BEANSBY B. COOPER, F. R. S., Senior Surgeon to Guy's Hospital, etc. ; p. 771, 8vo. Philadelphia, Blanchard & Lea.

THE press in this country has been prolific in books of Surgery. Within the last ten years we have had Operative Surgeon's Principles of Surgery, Lectures, Hand-books, Minor Surgery, Text-books, Systems, Elements, Monographs, etc. Many of these present points of excellence calculated to render them popular and useful works. The rapidity with which they have succeeded one another, has only kept pace with the changes of doctrines and the improvements and alterations which are daily taking place. Hence each new work has been hailed as an accession to surgical literature and an improvement on that which preceded it.

In this respect the works whose titles stand at the head of this page, may be praised as inculcating an improved pathology, and directing, in some instances, better methods of treatment. Miller's in particular, which is devoted to the pathology only of surgery, was favorably noticed in this Journal on the occasion of the issue of a former edition. The present is an improvement on that edition.

Prof. Pirrie's work is said to have been received with great favor in England, and has been generally praised by the medical press in this country.

Notwithstanding all these merits and successes, we think that we hazard nothing in asserting, that there is not in the English language, at the present time, a single work on Surgery which affords a tolerably fair view of the present state of the science. Such a work as should give a clear and concise view of the principles, with all the varieties of treatment and operations which have been proposed, and a just appreciation of each, is a desideratum. It may be said, that in the present state of our knowledge it would be impossible to bring such a work within reasonable limits, that it would be too voluminous to be read, if not too expensive to be purchased. This is erroneous; Boyer's Surgery, in eleven volumes, presented, in the early part of the present century, a sufficiently full account of the entire science, and has not to the present time been entirely superseded as a work of reference for the practitioner. Cooper's Surgical Dictionary filled a similar place in English literature at that time, and is still equal to any work in the language on many points.

There is no new English work to take the place of this. That the production of such a work is not impracticable is shown by the works of Vidal in French. This work, in five volumes, contains about the same amount of matter as Boyer's and is the kind of work demanded both by the student and practitioner. We have thought of presenting to our readers a chapter of this work, translated, in contrast with one on the same subject, from one of the works under notice, but we find that most of the subjects which would be interesting are not noticed at all in the latter.

A work on Surgery, such as we have described, would meet the wants of students as a "text" book, and of practitioners as a work of reference. An ordinarily industrious student will read during the winter time, all the articles on the subject of each day's lecture, in three or four of the most voluminous books on surgery in the language, without taking too much of his time. To limit his reading to a single "horn-book" or hand-book, as is too generally done, is a great error. The work which he needs is precisely that the practitioner wishes to refer to, viz.: one which gives a full view of each subject and the principal methods of all operations. Such a work need not be more voluminous than Cooper's Surgical Dictionary, many of the points formerly discussed at length being now settled, thus furnishing as much occasion for omitting old as for the introduction of new matter.

There is a chapter at the conclusion of Dr. Miller's work on Anæsthetics which, as embodying the opinions and the results of practice in Edinburgh, is worthy of attention. Dr. Miller, and the Surgeons and Accoucheurs of that city, prefer chloroform to ether. They administer it on a "handkerchief, lint, or sponge arranged after the fashion of a cone, saturated with chloroform, held at the distance of a few inches and gradually brought nearer until the mouth and nostrils are fairly inducted." As to the quantity used "the object is to produce insensibility as completely and as soon as we can, and there is no saying *a priori* whether this is to be accomplished by fifty drops or five hundred. We begin with generally two or three drachms spilt on a handkerchief or lint, and we refresh that or not as circumstances require." There are two points insisted on by Prof. Miller which are *essential* to the safe use of chloroform, and doubtless also of ether; these are, 1st, that the article should be pure; 2d, that its effects should be watched and its administration superintended by some experienced and competent person.

In regard to the purity, it appears that such an article as *pure* chloroform is entirely unknown in this market. Setting aside that often met with containing chlorine or muriatic acid, in a form absolutely irrespirable and poisonous, of which we have met with two specimens from good apothecaries, that directed by the Pharmacopœia contains, it seems, a portion of empyreumatic oil of exceedingly dangerous properties. Having recently used an article which had been subjected to an additional washing and distillation beyond that of the officinal process, we have noticed a perceptible difference in its action from that of the best we have before employed. Dr. Miller thinks its use diminishes the mortality of operations, and that it is only inadmissible in operations on the mouth and air passages, but should be used cautiously in cases of diseases of the heart and in hysterical subjects. In merit we rank 1st, Miller's; 2d, Pirrie's; 3d, Cooper's Lectures, and object to all of them, rather on the ground of imperfection than for any erroneous doctrines they contain. D. B.

